# Micafungin Elicits an Immunomodulatory Effect in *Galleria mellonella* and Mice

**DOI:** 10.1007/s11046-015-9940-z

**Published:** 2015-09-18

**Authors:** Beth Burgwyn Fuchs, Yan Li, Dedong Li, Tatiana Johnston, Gabriel Hendricks, Gang Li, Rajmohan Rajamuthiah, Eleftherios Mylonakis

**Affiliations:** Division of Infectious Diseases, Rhode Island Hospital, Alpert Medical School of Brown University, 593 Eddy Street, Aldrich 708, POB 328/330, Providence, RI USA; Pharmacy Department, Shandong Provincial Qianfoshan Hospital, Jinan, Shandong Province China; Department of Clinical Pharmacology, General Hospital of Chinese PLA, Beijing, China; The Miriam Hospital, Alpert Medical School of Brown University, Providence, RI USA; Department of Laboratory Medicine, Jinshan Hospital, Shanghai Medical College, Fudan University, Shanghai, China

**Keywords:** *Candida albicans*, Echinocandins, *Galleria mellonella*, Immunomodulatory, Micafungin

## Abstract

The echinocandin family of drugs is well characterized for antifungal function that inhibits β-d-glucan synthesis. The aim of this work was to study whether micafungin, a member of the echinocandin family, elicits additional activities that prime the host’s immune response. We found that in a *Galleriamellonella* model, prophylactic treatment with micafungin extended the life of *Staphylococcusaureus*-infected larvae (a pathogen to which the drug demonstrates no direct antimicrobial activity) compared to insects that did not receive micafungin (*P* < 0.05). The inhibition of pathogens in the *G*. *mellonella* infection model was characterized by a 2.43-fold increase in hemocyte density, compared to larvae inoculated with PBS. In a murine model where animals were provided micafungin prophylaxis 3 days prior to macrophage collection, macrophages were found associated with an average 0.9 more fungal cells per macrophage as compared to saline-treated animals. Interestingly, micafungin-stimulated macrophages killed 11.6 ± 6.2 % of fungal cells compared to 3.8 ± 2.4 % of macrophages from saline-treated animals. The prophylactic provision of micafungin prior to *Candida albicans* infection was characterized by an increase in the proinflammatory cytokines CXCL13 and *SPP1* by 11- and 6.9-fold, respectively. In conclusion, micafungin demonstrated the ability to stimulate phagocytic cells and promote an immune response that can inhibit microbial infections.

**Electronic supplementary material:**

The online version of this article (doi:10.1007/s11046-015-9940-z) contains supplementary material, which is available to authorized users.

## Introduction

The echinocandins are effectively used to treat fungal infections. Commercially available compounds in the echinocandins family include: caspofungin, micafungin, and anidulafungin. These compounds share a similar backbone with differing side chains. Echinocandins inhibit the glucan synthase protein FKS1, which produces (1,3)β-d-glucan, a major cell wall component that is conserved among fungi [1]. Glucan synthase genes are present in multiple alleles in various fungi encoded by the genes *FKS1*, *FKS2*, *and FKS3* from *Saccharomyces cerevisiae* and *GSL1* and *GSL2* from *Candida albicans* [2–4].

Further investigation of echinocandins has revealed that their role in preventing microbial infection may extend beyond that of direct antifungal activity. More specifically, the echinocandin caspofungin was shown to prime an immune response in a *Galleriamellonella* infection model [5]. These findings suggest that caspofungin elicits an immune response characterized by an increase in the number of circulating immune cells, and expression of humoral immune genes, including *IMPI* (inducible metalloproteinase inhibitor [6]), and transferrin [5], which can inhibit the microbial infection. *G*. *mellonellaIMPI* inhibits the metalloproteinases of bacterial pathogens [6], and as an associate with the immune system, transferrin impedes microbial survival by binding free iron [7].

The echinocandin micafungin has been shown to modulate an immune response. Moretti et al. [8] observed that micafungin could decrease the expression of tumor necrosis factor-α (TNF-α) and increase the expression of interleukin-10 (IL-10), while anti-inflammatory responses were dose dependent and functioned through IL-10 and required dectin-1. Further, echinocandins can influence immune responses by affecting the fungal cell wall integrity and exposing β-glucan that can elicit a PMN host response to the infecting fungi [9].

In this study, we use the *G*. *mellonella* infection model, explored in the work by Kelly et al. [5], to investigate whether another echinocandin, micafungin, can prime an immune response in *G*. *mellonella* and confirmed these findings using a mammalian model. Through our study, we find alterations to phagocytic cell responses in both model organisms.

## Materials and Methods

### Organisms and Strains

The microorganisms used in this study are listed in Table 1. *G*. *mellonella* were obtained from Vanderhorst Wholesale (St. Marys, Ohio). CD1 mice were acquired from Charles River Laboratories (Wilmington, MA).

**Table 1:** Microorganisms used in this study

Organism	Strain	Reference
*Staphylococcus aureus*	29213	ATCC
*Candida albicans*	CA36S	[10]
*Aspergillus fumigatus*	AF293	[24]

### *G*. *mellonella* Survival

Sixth-instar larvae were pretreated with 5 mg/kg of micafungin by injecting the compound at the last, left pro-leg. After 24 h, larvae were infected with 5 × 10^8^ cells/ml of *S*. *aureus* (strain ATCC 29213) in a volume of 10 μl. Ten larvae were used per infection group. PBS was included as a negative control and caspofungin as a positive control. Larvae were incubated at 37 °C and monitored daily for survival.

### Effects of Prophylactic Micafungin to *G*. *mellonella* Hemocyte Density

Larvae were pretreated with 5 mg/kg of micafungin by injecting the compound at the last, left pro-leg. Hemocytes were collected from the hemocoel at 4 h post-injection of micafungin. Larvae were bled into tubes containing cold sterile insect physiologic saline (IPS) (150 mM sodium chloride; 5 mM potassium chloride; 100 mM Tris–hydrochloride, pH 6.9 with 10 mM EDTA and 30 mM sodium citrate). The hemocytes were enumerated with the aid of a hemocytometer. Results were averaged from four replicates.

### Murine Infection Model

CD1, 6-week-old, female mice were prophylactically treated with 5 mg/kg micafungin daily for 3 days via peritoneal injection. A control group received saline daily. Subsequent to the prophylactic regimen (day 4), mice were infected with 1 × 10^6^ colony-forming units (CFU) *C*. *albicans* 36S (CA36S) [10] via tail vein injection. At the conclusion of the experiment, organs were harvested to evaluate the fungal burden (*n* = 6). All murine protocols were approved by the Rhode Island Hospital Institutional Animal Care and Use Committee.

### Labeling of Fungal Cells with Fluorescein-5-Isothiocyanate (FITC)

Overnight cultures of *C*. *albicans* CA36S or *A*spergillus *fumigatus* (AF293) grown at 30 °C with agitation were collected with centrifugation and washed twice with PBS. CA36S and AF293 were counted with a hemocytometer, and 10^6^ cells in PBS were incubated with 0.1 mg/ml FITC (Invitrogen, Molecular Probes, Waltham, MA) by adding 10 μl of 10 mg/ml FITC in DMSO to 990 μl of PBS. Cells were incubated for 30 min in the dark at room temperature. Cells were washed three times with PBS containing 1.5 % fetal bovine serum (FBS) [11].

### Macrophage Stimulation

CD1, 6-week-old, female mice were injected with 5 mg/kg/day micafungin intraperitoneally for 3 days. A control group of mice were injected with normal saline. At 24 h post-treatment with micafungin or saline (day 4 of the assay), mice were killed and alveolar and peritoneal macrophages were harvested for in vitro experiments. Washing the peritoneal cavity with 10 ml of ice-cold PBS enabled collection of peritoneal exudate cells. The cells were centrifuged (1400 rpm, 10 min, 4 °C) and suspended in RPMI 1640 with 10 % fetal bovine serum. The cells were then diluted to 1 × 10^5^ cells/ml and plated on chamber slides (Thermo Scientific, Lab-TekII) and incubated for 1 h to allow adherence to the glass surface. Monolayers of peritoneal phagocytes were incubated for 30 min at 37 °C in RPMI 1640 with 10 % fetal bovine serum containing 10 yeast per phagocyte. We used micafungin-resistant *C*. *albicans* CA36S stained by FITC before incubation. Post-incubation, trypan blue was added to quench the fluorescence of FITC fungal cells that were not engulfed by the macrophages. A total of 100 phagocytes were analyzed for each preparation, and the percentage of phagocytes, which had phagocytosed or attached *C*. *albicans*, and the mean number of blastoconidia cells per phagocytes were counted [12].

### Alveolar Macrophage Collection

After the mice were killed as described above, the trachea was then exposed and bronchoalveolar lavage was performed. Briefly, a 20-gauge Luer stub adapter attached to a 1-ml syringe was inserted into the trachea. Warm Hanks’ balanced salt solution (HBSS) with 1 mM EDTA and 100 μg/ml ampicillin was injected into the lung and recovered. The lavage was repeated three times and recovered fluid was pooled for assessment of cellular content. From each mouse, 1–3 × 10^5^ alveolar macrophages were obtained. The recovered cells were washed twice with PBS, and an aliquot of viable cells was set aside for further study.

### Macrophage Killing of Fungal Cells

1 × 10^5^ Exudate cells were plated (0.2 ml/well) in 24-well plates with lids and incubated with 1 × 10^6^ blastoconidia in 0.8 ml of RPMI 1640 containing 10 % fetal bovine serum for 30 min at 37 °C. Sterilized double-distilled water (1 ml) containing 0.5 % of Triton X-100 (Pharmacia, Stockholm, Sweden) was added to the wells for 10 min. Serial tenfold dilutions from each well were made in PBS. Aliquots (100 μl) were plated on YPD agar, and the number of CFU was determined after incubation for 24 h at 37 °C. The percentage of CFU reduction was compared between the micafungin group and control.

### Murine Infection and Peripheral Blood Gene Expression

CD1 mice were injected intraperitoneally with 5 mg/kg micafungin or saline for 3 consecutive days before infection. Two days before infection, CA36S was grown in liquid YPD at 30 °C with shaking overnight to late-log phase. The cells were centrifuged, washed in PBS, and suspended at a concentration of 1.5 × 10^7^ cells/ml. Mice were infected via the tail vein with 3 × 10^6^ cells of CA36S, in a volume of 200 µl. Mice were euthanized 24 h post-infection, and 100 µl of blood was collected from the heart and placed in RNAprotect animal blood tubes (Qiagen, Hilden, Germany). RNA was purified from the blood using RNeasy Protect Animal Blood (Qiagen, Hilden, Germany), and gene expression was determined with RT^2^ Profiler PCR Array PAMM-150ZD (Qiagen, Hilden, Germany) according to the manufacturer’s instructions.

### Statistical Analysis

STATA software was used to generate Kaplan–Meier survival curves. Student’s *t* test was used to compare hemocyte densities. A *P* value < 0.05 was considered significant.

## Results

### *Galleriamellonella* Infection Model

**Fig. 1:**
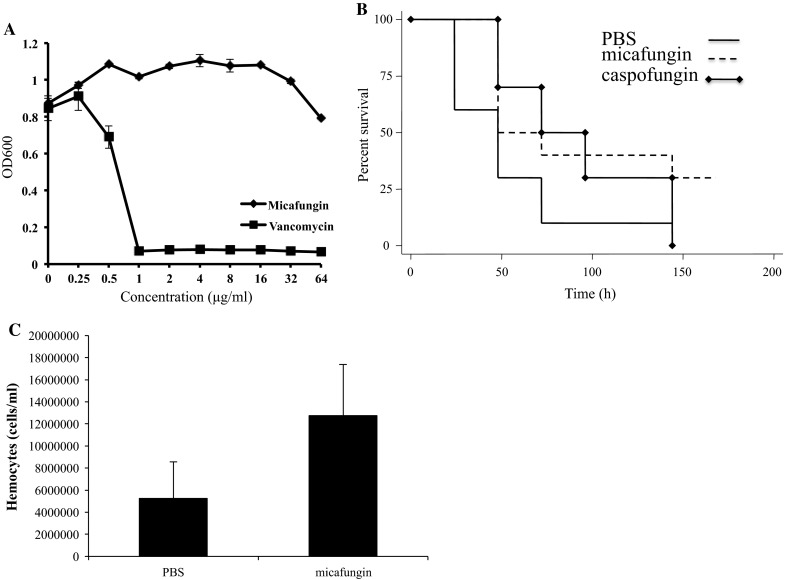
Micafungin prevents a lethal infection and establishes an immune response in *G*. *mellonella*. **a** *S*. *aureus* had a susceptible MIC to vancomycin but was resistant to micafungin. **b** *G*. *mellonella* larvae demonstrated enhanced survival to an *S*. *aureus* infection when prophylactically treated with echinocandins before the infection. **c** Larvae hemocyte density was increased with the provision of micafungin

In order to evaluate the hypothesis that micafungin may elicit an immunomodulatory effect in *G*. *mellonella*, we first evaluated whether micafungin can prolong survival after infection with *S. aureus*. Micafungin is an antifungal agent that does not inhibit bacteria, including *S. aureus*, and the minimal inhibitory concentration (MIC) of micafungin was >64 μg/ml against *S*. *aureus* (Fig. 1a). Although micafungin does not inhibit the bacteria directly, it does promote the survival of *G*. *mellonella*. More specifically, in this series of experiments, micafungin was injected 24 h prior to infection with *S*. *aureus*. Caspofungin prophylaxis treatment was included as a control since it has previously been shown to promote survival of *S*. *aureus*-infected larvae [5]. Both compounds contributed to significantly prolonged survival compared to larvae that received PBS rather than prophylaxis treatment (*P* < 0.05; Fig. 1b). Although it does not demonstrate a direct antibacterial effect against *S. aureus,* micafungin promoted survival of *G*. *mellonella* infected with *S*. *aureus*.

Therefore, we investigated micafungin’s ability to prime the *G*. *mellonella* immune response. Immune responses of *G*. *mellonella* can be primed through changes to gene expression or the number of available hemocytes, the phagocytic cells of the larvae hemolymph, which include: prohemocytes, caogulocytes, spherulocytes, oenocytoids, plasmatocytes, and granulocytes [13]. We tested whether micafungin exposure affects hemocyte. At 4 h post-injection with micafungin, hemocyte density increased on average 7.5 × 10^6^ more cells/ml compared to a PBS control (*P* < 0.05; Fig. 1c), indicating that micafungin recruits hemocytes into the hemolymph. The hemocyte density decreased over time and while it was 1.3-fold higher in micafungin-treated larva 24-h after injection, the increase was not significant over PBS-injected larva (data not shown). Thus, micafungin appears to cause an immediate increase in the number of available hemocytes, recruiting the phagocytic cells to the hemocoel. Of note, our experiment analysis did not differentiate between the 6 different types of hemocytes within the hemolymph.

### Murine Infection Model

**Fig. 2:**
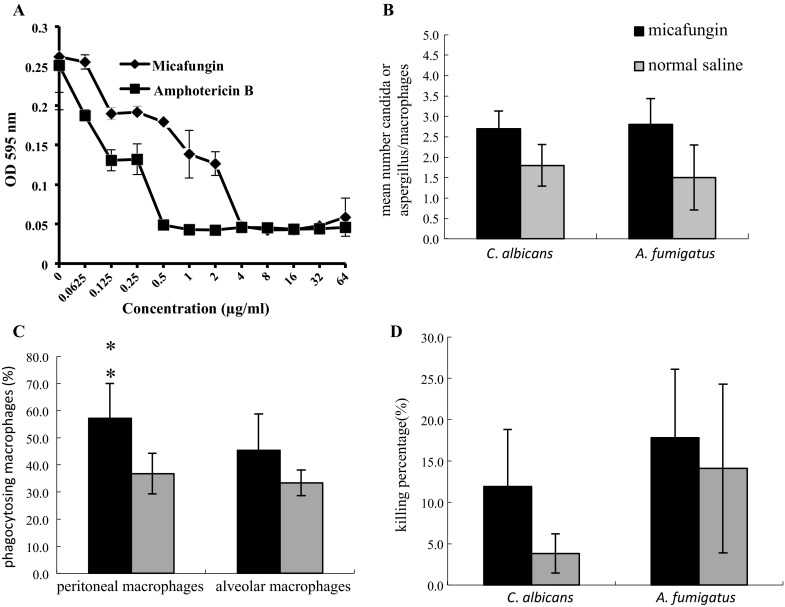
Micafungin treatment stimulates phagocytoses of *C*. *albicans* and *A*. *fumigatus*. **a** The CA36S strain used to infect the mice was tolerant to micafungin, exhibiting an MIC of 4 μg/ml. **b** Mice were treated with micafungin to determine whether macrophages were stimulated to enhance phagocytosis of *C*. *albicans* or *A*. *fumigatus*. After treatment with micafungin or saline, peritoneal and alveolar macrophages were collected and exposed to fungus to evaluate difference in phagocytosis. Peritoneal macrophages were able to phagocytose the CA36S significantly better when stimulated with micafungin compared to saline. **c** Macrophages that were stimulated associate with fungal cells, either *C*. *albicans* or *A*. *fumigatus*. **d** The stimulated macrophages were able to kill fungal cells, *C*. *albicans* were more vulnerable to killing by peritoneal macrophages than *A*. *fumigatus*

To determine whether the immune stimulation elicited by micafungin extended to a mammalian system, we investigated the effect of micafungin treatment on a *C*. *albicans* infection in mice. We infected mice with a micafungin-resistant strain, CA36S, that has a mutation in *FKS1* and exhibits an MIC of 4 μg/ml [10] (Fig. 2a). There was no increase in survival or a reduction in fungal burden after administration of 5 mg/kg micafungin for 3 days prior to infection (data not shown).

Although survival was not prolonged based on our dosing regimen, we investigated to determine whether phagocytic cells were stimulated, stemming from our findings that hemocyte density was augmented by micafungin exposure in the *G*. *mellonella* model. Indeed, we found that treatment of mice with micafungin promoted the phagocytosis of CA36S and *A*. *fumigatus* by alveolar macrophages as compared to macrophages isolated from mice treated with saline (*P* = 0.01; Fig. 2b). By counter-staining with trypan blue, FITC-stained cells not phagocytosed were quenched, leaving only cells within the macrophages to be visualized by FITC. We observed a greater number of fungal cells associated with macrophages from micafungin-treated mice as compared to macrophages from saline-treated mice [2.7 ± 0.4 *C*. *albicans* cells per macrophage versus 1.8 ± 0.5 *C*. *albicans* cells per macrophage with saline treatment (*P* > 0.05)] (Fig. 2b). The association with a greater number of fungal cells per macrophages was also observed when *A.fumigatus* was introduced as a pathogen to micafungin-stimulated cells compared to saline (*P* > 0.05; Fig. 2b). Among the 100 peritoneal macrophages observed for each group, 57.2 % were phagocytosing *C*. *albicans* in the micafungin group, compared to 36.8 % from the saline treatment group (Fig. 2c). The increase was also true for alveolar macrophages, 45.4 % of micafungin-pretreated cells had phagocytosed *C*. *albicans* cells, compared to 33.4 % macrophages pretreated with saline, although statistical significance was not reached among alveolar macrophages.

To test the overall killing ability of macrophages from mice treated with micafungin, we measured the number of CFU available in a culture after stimulated macrophages were allowed to incubate with fungal cells. We determined that micafungin-exposed macrophages were able to kill fungal cells better than macrophages derived from saline-treated mice (*P* > 0.05; Fig. 2d). Micafungin-stimulated macrophages killed 11.6 ± 6.2 %, compared to 3.8 ± 2.4 % of macrophages from saline-treated animals. Thus, micafungin appears to stimulate the macrophage response enabling better association and killing of the fungal cells.

**Fig. 3:**
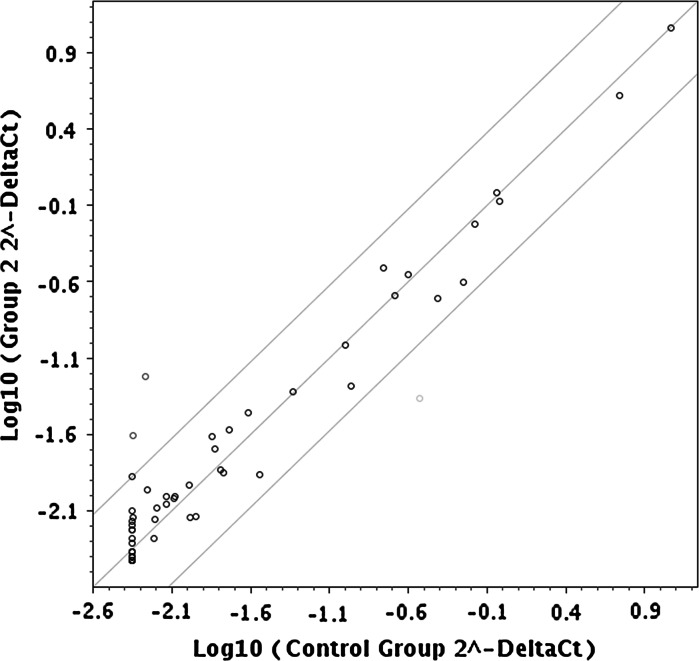
Prophylaxis with micafungin elicits specific response directed at B cell migration. Gene expression was compared in blood collected from animals prophylactically treated with micafungin or saline prior to *C*. *albicans* infection. Investigation of a panel of chemokines and cytokines showed increased expression of two genes and reduced expression of one gene

Using RT^2^ Profiler PCR Array mouse cytokines and chemokines, PAMM-150ZD, the change in gene expression was evaluated to determine whether micafungin elicited an immune response. Expression was normalized to actin. We compared the differences of gene regulation between mice pretreated with micafungin and then infected with CA36S to animals pretreated with saline before infection with CA36S. From this comparison, we found two genes that exhibited increased expression, CXCL13 and *SPP1* (Fig. 3). An 11-fold increased expression of CXCL13 (*BCA1*), a c-x-c motif chemokine ligand 13, was coupled with a fivefold increase in *SPP1*, secreted phosphoprotein. The increased expression of both of these genes could suggest a recruitment of B cells in response to micafungin. We found that *platelet factor*-*4* (*PF4*), also a c-x-c chemokine, known as CXCL4, was downregulated 6.9-fold. PF4 plays a role in wound repair and inflammation. Results from the full panel of genes evaluated are available in Supplemental Table 1. The results suggest that micafungin is indeed stimulating an immune response that could obstruct a microbial infection.

## Discussion

Our study investigates the response of phagocytic cells upon introduction of micafungin and how this aids to inhibit a microbial infection. We found increased phagocytic activity by macrophages and greater number of associations between fungal cells and phagocytic cells in both an insect and a mammalian model.

The immune response elicited by the insect *G*. *mellonella* can be used as a comparison to the innate immune response of mammals. Both organisms have structures/barriers to obstruct invading pathogens and cellular responses to breaching microbes. In both organisms, phagocytic cells respond to invading pathogens once they enter the organism. A possible mechanism to manipulate this response as a means of prophylactic treatment for infections is to prime the immune response, particularly phagocytic cells, so they can attend to invading pathogens in susceptible patients.

The echinocandin caspofungin was previously shown to promote an immune response in *G*. *mellonella* that improved the survival of larvae infected with pathogens, to which the compound shows no direct inhibitory activity [5]. Caspofungin not only promoted the survival of *G*. *mellonella* larvae infected with *S*. *aureus*, a pathogen it does not inhibit in vitro, but also was found to increase the hemocyte density [5]. This was accomplished through the increased expression of *IMP1* and transferrin, part of the antimicrobial arsenal, and was accompanied by an increase in the number of hemocytes [5].

Hemocytes are the collection of phagocytic cells in *G*. *mellonella*. Like mammalian phagocytic cells, hemocytes respond to invasive agents. When pathogenic microbes breach the larvae cuticle, the number of hemocytes does not increase; however, in response to less pathogenic microbes, the hemocyte density increases [14]. The hemocyte density can also be primed for a short period (24 h) by heat or physical stress conditions that protect the larva from infection [15], thus indicating that hemocytes are made available when the larvae are able to combat the pathogen and establishing an inverse relationship with hemocyte density and microbial pathogenicity [14]. Overall, *G*. *mellonella* hemocyte density is a malleable defense attribute that responds to the presence of pathogens or immunomodulating compounds. A higher hemocyte density correlates to better outcomes after infection.

Our study found that phagocytic cells were activated in murine assays as well, noted by the increased association with and killing of fungal cells by macrophages, particularly by peritoneal macrophages. Our investigation to determine whether the defense genes were harnessed by micafungin to elicit a response identified two candidates, CXCL13 and *SPP1*, both exhibiting fold increases in the presence of micafungin.

The chemokine CXCL13, the ligand for CXCR5 (*BLR1*), is a B cell chemoattractant. CXCL13 also guides follicular B helper T cells and dendritic cells. It is expressed in liver, spleen, lymph nodes, appendix, and stomach as a homeostatic functional group [16]. CXCL13 was also found to be expressed in Paneth cells, an epithelial subset of cells specialized in antimicrobial peptide production (AMP), of Macaque [17]. Sodium butyrate, a short-chain fatty acid that is a product of bacterial metabolism, inhibits histone deacetylase activity and increases the expression of CXCL13 in a Paneth cell line [17]. In an assay testing the antimicrobial properties of a single concentration of CXCL13, it was found to have inhibitory activity killing 83 and 50 %, *E*. *coli* and *S*. *aureus*, respectively [18].

The additional finding that *SPP1* was expressed after treatment with micafungin contributes to the indications that micafungin induces the recruitment of monocytes. *SPP1*, secreted phosphoprotein 1, also known as osteopontin (OPN), exhibited increased expression in animals treated with micafungin. OPN is secreted by activated T and B cells, NK cells, dendritic cells, and macrophages [19, 20]. Macrophages play a role in innate and adaptive immune responses; the degree of the response in both categories is influenced by the expression of OPN and has been shown to bind to and opsonize bacteria for phagocytosis [19, 21]. OPN forms a gradient that chemotactically attracts monocytes [21]. Thus, playing a role to provide resistance to infection. OPN-knockout mice have compromised immunity and fail to respond to or clear microbial infections [22, 23].

Our data suggest that micafungin primes the immune response to microbial infection, even to pathogens that it does not directly inhibit. Combined, these responses enable macrophages to better inhibit the invading pathogen. It is notable that CXCL13, characterized to be expressed in Paneth cells specific to the epithelial crypt, was identified and that we find increased activity of macrophages derived from the peritoneal cavity compared to alveolar macrophages. Perhaps there is signaling in the gut that can be manipulated since this is often the first site of microbial entry or colonization for organisms and where bacteria must be managed. This report lends support to data showing a role for echinocandins to direct macrophages to respond to invading microbes evidenced by both caspofungin [5] and micafungin activation of phagocytic cells.

## Electronic supplementary material

Supplementary material 1 (DOCX 29 kb)
