# Supplementary material for: Micafungin Elicits an Immunomodulatory Effect in Galleria mellonella and Mice
Source: Mycopathologia. 2015 Sep 18;181:17–25. doi: 10.1007/s11046-015-9940-z (PMC4676791; doi:10.1007/s11046-015-9940-z)
Supplement: Supplementary file 1 — Supplementary material 1 (DOCX 29 kb) [file 11046_2015_9940_MOESM1_ESM.docx]

| s**upplemental Table 1. Panel of Immune Response Genes** | | |
| --- | --- | --- |
| **Symbol** | **Description** | **Fold Change compared to control** |
| Adipoq | Adiponectin, C1Q and collagen domain containing | 0.9582 |
| Bmp2 | Bone morphogenetic protein 2 | 0.9012 |
| Bmp4 | Bone morphogenetic protein 4 | 0.8476 |
| Bmp6 | Bone morphogenetic protein 6 | 0.9016 |
| Bmp7 | Bone morphogenetic protein 7 | 0.8452 |
| Ccl1 | Chemokine (C-C motif) ligand 1 | 0.8452 |
| Ccl11 | Chemokine (C-C motif) ligand 11 | 0.8466 |
| Ccl12 | Chemokine (C-C motif) ligand 12 | 1.1795 |
| Ccl17 | Chemokine (C-C motif) ligand 17 | 0.9656 |
| Ccl19 | Chemokine (C-C motif) ligand 19 | 1.5846 |
| Ccl2 | Chemokine (C-C motif) ligand 2 | 1.526 |
| Ccl20 | Chemokine (C-C motif) ligand 20 | 0.8452 |
| Ccl22 | Chemokine (C-C motif) ligand 22 | 0.896 |
| Ccl24 | Chemokine (C-C motif) ligand 24 | 0.8452 |
| Ccl3 | Chemokine (C-C motif) ligand 3 | 1.3412 |
| Ccl4 | Chemokine (C-C motif) ligand 4 | 1.4323 |
| Ccl5 | Chemokine (C-C motif) ligand 5 | 1.7731 |
| Ccl7 | Chemokine (C-C motif) ligand 7 | 1.3412 |
| Cd40lg | CD40 ligand | 1.1939 |
| Cd70 | CD70 antigen | 0.8452 |
| Cntf | Ciliary neurotrophic factor | 1.1215 |
| Csf1 | Colony stimulating factor 1 (macrophage) | 0.8332 |
| Csf2 | Colony stimulating factor 2 (granulocyte-macrophage) | 0.8452 |
| Csf3 | Colony stimulating factor 3 (granulocyte) | 0.8452 |
| Ctf1 | Cardiotrophin 1 | 0.8452 |
| Cx3cl1 | Chemokine (C-X3-C motif) ligand 1 | 0.8452 |
| Cxcl1 | Chemokine (C-X-C motif) ligand 1 | 0.8452 |
| Cxcl10 | Chemokine (C-X-C motif) ligand 10 | 0.4791 |
| Cxcl11 | Chemokine (C-X-C motif) ligand 11 | 0.8452 |
| Cxcl12 | Chemokine (C-X-C motif) ligand 12 | 2.9834 |
| Cxcl13 | Chemokine (C-X-C motif) ligand 13 | 11.039 |
| Cxcl16 | Chemokine (C-X-C motif) ligand 16 | 1.9339 |
| Cxcl3 | Chemokine (C-X-C motif) ligand 3 | 0.8452 |
| Cxcl5 | Chemokine (C-X-C motif) ligand 5 | 0.8452 |
| Cxcl9 | Chemokine (C-X-C motif) ligand 9 | 0.8452 |
| Fasl | Fas ligand (TNF superfamily, member 6) | 1.1802 |
| Gpi1 | Glucose phosphate isomerase 1 | 1.1078 |
| Hc | Hemolytic complement | 0.8452 |
| Ifna2 | Interferon alpha 2 | 0.8452 |
| Ifng | Interferon gamma | 0.6527 |
| Il10 | Interleukin 10 | 1.4346 |
| Il11 | Interleukin 11 | 0.8452 |
| Il12a | Interleukin 12A | 1.3372 |
| Il12b | Interleukin 12B | 0.8452 |
| Il13 | Interleukin 13 | 0.8452 |
| Il15 | Interleukin 15 | 0.6842 |
| Il16 | Interleukin 16 | 0.9561 |
| Il17a | Interleukin 17A | 0.8452 |
| Il17f | Interleukin 17F | 0.8452 |
| Il18 | Interleukin 18 | 1.1345 |
| Il1a | Interleukin 1 alpha | 0.8452 |
| Il1b | Interleukin 1 beta | 0.442 |
| Il1rn | Interleukin 1 receptor antagonist | 0.4809 |
| Il2 | Interleukin 2 | 0.8452 |
| Il21 | Interleukin 21 | 0.8452 |
| Il22 | Interleukin 22 | 0.8452 |
| Il23a | Interleukin 23, alpha subunit p19 | 0.8452 |
| Il24 | Interleukin 24 | 0.8452 |
| Il27 | Interleukin 27 | 1.1681 |
| Il3 | Interleukin 3 | 0.8452 |
| Il4 | Interleukin 4 | 1.095 |
| Il5 | Interleukin 5 | 0.8452 |
| Il6 | Interleukin 6 | 0.8863 |
| Il7 | Interleukin 7 | 0.8452 |
| Il9 | Interleukin 9 | 0.8452 |
| Lif | Leukemia inhibitory factor | 0.8452 |
| Lta | Lymphotoxin A | 0.9618 |
| Ltb | Lymphotoxin B | 0.9014 |
| Mif | Macrophage migration inhibitory factor | 0.9917 |
| Mstn | Myostatin | 0.8452 |
| Nodal | Nodal | 0.8452 |
| Osm | Oncostatin M | 1.684 |
| Pf4 | Platelet factor 4 | 0.1448 |
| Ppbp | Pro-platelet basic protein | 0.509 |
| Spp1 | Secreted phosphoprotein 1 | 5.4539 |
| Tgfb2 | Transforming growth factor, beta 2 | 1.3044 |
| Thpo | Thrombopoietin | 0.8452 |
| Tnf | Tumor necrosis factor | 0.9065 |
| Tnfrsf11b | Tumor necrosis factor receptor superfamily, member 11b (osteoprotegerin) | 0.8452 |
| Tnfsf10 | Tumor necrosis factor (ligand) superfamily, member 10 | 1.783 |
| Tnfsf11 | Tumor necrosis factor (ligand) superfamily, member 11 | 0.8452 |
| Tnfsf13b | Tumor necrosis factor (ligand) superfamily, member 13b | 0.8603 |
| Vegfa | Vascular endothelial growth factor A | 1.347 |
| Xcl1 | Chemokine (C motif) ligand 1 | 1.0185 |
